# Prevalence of Homelessness in the Emergency Department Setting

**DOI:** 10.5811/westjem.2017.1.33054

**Published:** 2017-03-07

**Authors:** Brett J. Feldman, Cristina G. Calogero, Kareem S. Elsayed, Osman Z. Abbasi, Joshua Enyart, Timothy J. Friel, Yasir H. Abunamous, Stephen W. Dusza, Marna Rayl Greenberg

**Affiliations:** * Lehigh Valley Hospital and Health Network/USF MCOM CC & I-78, Department of Medicine, Allentown, Pennsylvania; † Lehigh Valley Hospital and Health Network/USF MCOM CC & I-78, Department of Emergency Medicine, Allentown, Pennsylvania; ‡ Lehigh Valley Hospital and Health Network/USF MCOM CC & I-78, Department of Family Medicine, Allentown, Pennsylvania

## Abstract

**Introduction:**

According to the National Alliance to End Homelessness, the national rate of homelessness has been cited as 17.7 homeless people/10,000 people in the general population, and 24.8 homeless veterans/10,000 veterans in the general population. However, it is unknown what the prevalence of homelessness is in the emergency department (ED) setting. We set out to determine the prevalence of homelessness or at risk for homelessness in the ED setting.

**Methods:**

Using a five-question screening tool derived from the U.S. Department of Housing and Urban Development, Health and Human Services and the Veterans Administration definition for homelessness, we surveyed all patients meeting inclusion/exclusion criteria on scheduled shifts in one of three EDs in Northeastern Pennsylvania. To participate, subjects had to be a registered patient in the ED, be 18 years or older, speak English, have the capacity to answer survey questions, not be critically ill, be willing to participate, and not have taken the survey before. We selected two survey periods to represent seasonal variations.

**Results:**

We included 4,395 subjects in the analysis. The mean age of those who screened positive for homelessness or at risk for homelessness was 43.1 (SD 16.6). Overall, 136 (3.1%) participants screened positive for at risk for homelessness and 309 (7.0%) screened positive for homelessness. A total of 103 subjects (9.8%) screened positive for homelessness or at risk for homelessness on weekends and 312 (10.3%) on weekdays (p=0.64). The proportion of those screening positive for homelessness or at risk for homelessness varied by site: 145 (7.5%) at the trauma center, 151(9.1%) at the suburban site, and 149 (18.7%) at the center city site, p<0.001.There was no statistical significance to the difference between the trauma center and the suburban site (p=.088), but there was statistical significance between both the suburban and the trauma center when compared to the center city site (both p<0.0001). The proportion of those screening positive for homelessness in the summer months (156, 7.5%) was similar to those in the winter months (153, 6.6%), p=0.23.,

**Conclusion:**

In our study, the overall prevalence of homelessness or at risk for homelessness was 10.1 percent. This prevalence did not seem to vary between weekdays and weekends. Additionally, summer months had a prevalence that was as concerning as winter months. The prevalence does, however, seem to vary by institutional characteristics even in the same geographic region. Understanding the patterns of prevalence of homelessness is a step toward considering possible interventions to assist this vulnerable population.

## INTRODUCTION

Nearly 1.5 million Americans spend at least one night in an emergency shelter or transitional housing each year, and on any given night in the U,S. over 500,000 people are homeless.^1^ Homeless people have substantially higher rates of emergency department (ED) and hospital use compared to the general population.^2^, ^3^ Homeless people suffer from serious medical conditions and when hospitalized have longer lengths of stay than patients who are not homeless, which results in excess medical costs.^3^ Lack of a common definition of homelessness and our healthcare system’s inability for early identification and documentation of homeless patients are barriers to adequately assessing the extent of the problem and subsequent proper care.^4^,^5^

Homeless patients in the ED may not be easily identifiable on chart review because the patient might often list the address of a shelter, a friend or family member’s house, or a fictitious address as their primary residence.^6^ The prevalence of homelessness typically cited is usually generated from queries of national databases that rely on self-reporting of homelessness, or data from local shelters to identify the burden of homelessness, which may not adequately assess the issue.^6^–^8^ For example, studies from different time periods that used the National Hospital Ambulatory Care Surveys (NHAMCS-ED) reported only .4%– .6% of the “patient residence” data entries were listed as “homeless.”^6^,^9^ One study in the emergency medicine (EM) setting found that EM trainees often relied on visual pattern recognition to identify homeless patients, introducing a type of bias and creating an unreliable way to identify this population.^10^

The optimum way to determine the prevalence of homelessness in the ED setting has not been determined, and thus the magnitude of this problem has not been clearly defined. This is also complicated by resistance by the homeless to self-identify.^11^ The existing literature on this topic has proffered and advocated for universal screening as a route to address the medical and social needs of patients who are homeless.^9^ We set out to determine the prevalence of homelessness in the ED setting and to explore whether the prevalence varied between seasons (summer and winter) and between weekdays and weekends.

## METHODS

After the institutional review board expedited review and approval, a prospective survey was administered in three EDs in northeastern Pennsylvania. The contributing network hospitals were an inner city hospital with an annual census of over 20,000 visits per year (Site A), a Level I suburban trauma center with an annual census of 100,000 visits per year (Site B), and a suburban hospital with an annual census of 45,000 visits per year (Site C). Site characteristics (payer mix and admission rates) are listed in Table 1.

**Population Health Research Capsule**

What do we already know about this issue?

Homeless people have substantially higher rates of ED and hospital use compared to the general population. However, it is unknown what the prevalence of homelessness is in the ED setting.

What was the research question?

We set out to determine the prevalence of homelessness or at risk for homelessness in the ED setting.

What was the major finding of the study?

The prevalence of homelessness or at risk for homelessness was 10.1% and it did not vary between weekdays/weekends or season.

How does this improve population health?

Understanding the patterns of prevalence of homelessness is a step toward considering possible interventions to assist this vulnerable population.

To participate, subjects had to be a registered patient in the ED, be 18 years or older, speak English, have the capacity to answer survey questions, not be critically ill, be willing to participate, and not have taken the survey before. A five-question screening tool was derived from the U.S. Department of Housing and Urban Development (HUD), Health and Human Services (HHS) and the Veterans Administration (VA) definition for homelessness.^12^–^14^ In order to be used as a discriminatory point on the screening tool for homelessness, the qualification had to be present in at least two of the three definitions. The screening tool consisted of five “Yes” or “No” questions. The first question was a self-identifying question for “at risk” for homelessness, and the remainder of the questions (2–5) were used to screen for homelessness. Specifically subjects were asked, “In the last 60 days have you—”:

1. Been concerned about losing your housing
2. Changed residences more than twice*^+^
3. Lived with a friend or family member you do not normally reside with due to financial hardship^^+^
4. Been evicted or served an eviction notice*^+^
5. Slept outside, in an abandoned building, your car, in an emergency shelter, or in a motel due to financial hardship. *^^+^

(* derived from HUD, ^ derived from HHS, ^+^ derived from VA definition[s])

To improve validity, we tested the tool at site A using a convenience sample of patients over a period of four weeks (N=28). In response to feedback from these encounters, minor word changes to allow for better comprehension and reordering of the questions occurred. These results were not included in the study data. Thereafter, the study began and all eligible patients presenting to the ED were approached for study participation on systematically scheduled shifts in each of the three network EDs.

Shifts (either A.M. or P.M.) were the same hours at all three sites and were selected to proportionately represent site location, and evenly represent time of day, and day of the week. Site A had one pod (an area of defined beds cared for by an assigned physician), Site B had four pods, and Site 3 had three pods. Therein, sites with higher volume census have more pods and thus had more data hours for collection represented in the sample. By convenience, survey time periods were chosen to ensure representation of both summer and winter months (May 27–August 6, 2015, and December 3, 2015–February 29, 2016) and to capture seasonal variation. Surveys were administered by study team members (residents and students) who were not blinded to the study goal of determining homelessness prevalence. The primary outcome was the prevalence of homelessness or at risk for homelessness in the ED setting.

### Analysis

The survey responses were coded positive for homelessness if subjects responded “yes” to the questions related to changing a residence more than twice, living with a friend of family member, having been evicted or served an eviction notice, or having slept outside or in an abandoned building, car or motel due to financial hardship. Respondents were considered “at risk” for homelessness if they positively responded to the question related to concern about losing housing. Participants who responded positively to the “at risk” question and positively to any of the “homeless” questions were considered homeless, not “at risk.”

We summarized the categorical parameters of clinical enrollment site, season (winter versus summer), and time of week (weekday versus weekend) as a proportion of the subject group. Comparisons of the distribution of homelessness or at risk of homelessness by other study variables were made using chi-square. We used logistic regression to assess the association between survey questions and 1) clinical enrollment site, 2) weekday-versus-weekend survey administration, and 3) seasonality. For all models, clinical enrollment “Site B” was used as the referent, since it had the largest enrollment of the three contributing sites. Logistic models incorporated respondent sex and age to help control for potential confounding. We performed all analyses using Stata software v.14.1 (Stata Corporation, College Station, TX).

## RESULTS

A total of 7,232 patients were approached for study enrollment. Of these, 2,738 (37.9%) did not participate. The leading reasons for non-participation were that the patient 1) did not meet age requirement (n=847, 31%); 2) did not have capacity (n=654, 24%); 3) refused or not interested (n=350, 13%); 4) did not speak English (n=340, 12%); or 5) was critically ill (n=275, 10%). A total of 4,494 patient evaluations were completed between May 27, 2015, and February 29,2016, on 150 separate screening dates. Of these, we excluded 99 evaluations due to a respondent reporting taking the survey at an earlier date. The remaining 4,395 evaluations were included in the analysis. A majority of the respondents were female (58.2% n=2,557) and the average participant age was 50.8 years (SD=20.5). After excluding the patients who did not meet eligibility, those who participated in the survey were more likely to be female (63.7% versus 60.1%, p=0.002), older (55.6 versus 50.8 years, p<0.001) and enrolled at Sites A and B, compared to Site C (69.5% and 65.3% versus 45.7%, respectively, p<0.001).

The 4,395 participant evaluations occurred at three different clinical enrollment sites. The plurality of the surveys (n=1,939, 44.1%) were completed at Site B (trauma center) while Site C (suburban hospital) had 1,663 (37.8%) and Site A (inner city hospital) had the fewest (n=793, 18.0%) (Table 2). Participant characteristics differed between enrollment sites. On average, Site A had younger participants, with a mean age of 39.1 (SD=15.6) years, compared to 54.7 (SD=20.8) years for Site B (p<0.001) and 51.8 (SD=20.1) years for Site C (p<0.001). Modest differences in gender distribution were also noted between facilities with 62.8% of Site A respondents being female, compared to 55.4% and 59.2% for Site B (p<0.001) and Site C (p=0.09), respectively. Overall, 10.1% (n=445) of the survey respondents were homeless or at risk of homelessness. The prevalence of homelessness or being at risk differed between clinical enrollment sites. With Site B being the referent population, respondents at Site A were 2.9 times more likely to report being at risk or being homeless (OR=2.9; 95%, confidence interval [CI] [2.2–3.7]).

Responses for the individual screening questions are presented in Table 3. Overall 5.8% (n=255) of participants reported “being concerned about losing their home,” while 5% (n=221) reported living with a family member or friend. Fewer respondents reported a change in residence (n=75, 1.7%), being evicted or being served eviction papers (n=66, 1.5%) or sleeping outside or in an abandoned building (n=81, 1.8%).

After controlling for age and gender, we observed significant differences in response between enrollment sites, with participants at Site A (inner city) consistently reporting affirmative responses to each of the five survey questions. With Site B (trauma center) as a referent, participants from Site A were 2.7 times more likely to report changing their address (OR=2.7; 95% CI [1.5–4.8]), 2.3 times more likely to report being concerned about losing their home (OR=2.3; 95% CI [1.7–3.2]), 2.8 times more likely to report living with a family member or friend (OR=2.8; 95% CI [2.0–4.0]), 3.0 times more likely to report being evicted (OR=3.0; 95% CI [1.6–5.7]), and 3.1 times more likely to report having slept outside or in an abandoned building (OR=3.1; 95% CI [1.8–5.4]).

The timing of survey administration is presented in Tables 4 and 5. Overall, 69.1% of the surveys were administered on weekdays. We observed no significant differences in the distribution of survey responses between weekday and weekend administration. Overall, 52.8% of surveys (n=2321) were administered in the winter and 47.2% (n=2074) in the summer. Of the 5.8% of the sample who reported being concerned about losing their housing, no significant difference was observed by the season of survey administration with 6.5% of respondents reporting concern in the summer and 5.2% reporting concern in the winter months (OR= 1.2; 95% CI [0.9–1.5]).

## DISCUSSION

Lack of a standardized definition for homelessness across medical specialties and settings has been a barrier to the recognition and care of impacted patients.^5^ Proper identification of this vulnerable population needs to begin somewhere, and accurate screening in the ED could become an important setting for early interventions.^10^ In our study we found the prevalence range of at risk of homelessness or homelessness to vary from 7.5% to 18.8% based on site variability with the urban site having the highest prevalence. This range seemed higher than authors anticipated in context of the prior studies based on NHAMCS-ED data and in context that the national rate of homelessness has been cited as 17.7 homeless people per 10,000 people in the general population, and 24.8 homeless veterans per 10,000 veterans in the general population.^6^.^9^, ^15^

Although the prevalence of homeless patients was higher at the inner city ED (Site A), the trauma center (Site B) and the suburban (Site C) sites both had a higher homelessness prevalence than authors might have perceived, dispelling our own stereotype that homeless patients only present to inner-city facilities. The difference between the positive homeless responses and the season of the year also dispels any potential misunderstanding that homelessness is only an issue during the colder winter months when patients have no warm-shelter provisions. This is also consistent with a study done in England, which found no evidence to suggest that homeless people are more likely to attend the ED in cold weather and actually found a small positive correlation between rate of attendances and daily temperature, somewhat consistent with what our data shows.^16^

Additional implications can be drawn from the lack of statistically significant difference between homeless responses on weekdays or weekends. While there is an abundance of literature about homelessness in the ED, prior work has been more focused on our role as providers, the relationship of homelessness and frequent utilization of resources, excess cost of care for the homeless, and addressing the medical and social needs of the homeless, while our study is unique in its goal of determining prevalence in different ED settings (both urban and suburban). 2–4, 6, 8, 10 As our results demonstrate, homelessness is a concern for healthcare providers year round, regardless of the season, site or day of week. These results provide insight into the prevalence of homelessness in the ED, and contribute to future decisions about the allocation of resources to assist in the care of this population.

In our study, subjects with positive screening for homelessness or “at risk for homelessness” were offered a street medicine consultation. This consult team provides care for the homeless population using an interdisciplinary mobile team approach (physician assistants, doctors, nurses, financial aid planners, etc). They are available for outpatient and inpatient consultations at all three sites and are funded by grants, private donors and institutional support. Of note, consultants anecdotally reported getting engaged earlier in the patients’ care if they were admitted (Day zero), and consults placed based on positive screenings during the study time period were all deemed appropriate by the consulting service.

Future research is needed on what benefits detection of homelessness using this screening protocol provides. A cost analysis is vital, especially since our specialty is already overburdened with screening requirements (substance use, domestic violence, fall risk, etc.). Factored into the cost of screening must be actual patient outcomes, the potential money saved in the healthcare system, and at homeless shelters and the many other factors impacted by homelessness. The benefits of implementing this type of universal screening for homelessness in the ED setting must be considered in context of the potential cost savings.

## LIMITATIONS

These findings may not be geographically generalizable to other ED populations, although the survey was administered in both urban and suburban settings. Additionally, our coverage area has about 120 permanent emergency shelter beds for males and 22 for women for about 250,000 people in the region. There is no national database to describe how these shelter-bed resources compare to other geographic region, and it is unknown what impact that may have had on our results. The eligibility requirements (particularly the requirement of speaking English) may have caused selection bias. Other sampling issues must be considered when interpreting our results (total subjects eligible, schedules that were applied, the study period selected, the disproportionate responses from each of the sites and the potential impact on the accuracy of the data). Participant’s race was not collected as a part of the survey and its impact as a confounder to site variability is not known.

The survey was based on predetermined definitions of homelessness, but it has not been evaluated or strictly validated. Homeless people constitute a rare and elusive population, and effectively quantifying this population is made more difficult by the absence of an agreed-upon definition across time and place. This lack of definition results in a bias or unreliability in counting.^17^ Virtually all definitions require enumerators to make a decision as to whether the person is homeless according to operationalized measurement definition. 8

## CONCLUSION

In our study, the overall prevalence of homelessness or at risk for homelessness was over 10%. This prevalence did not seem to vary between weekdays and weekends or by season as summer months had a prevalence that was as concerning as winter months. The prevalence does, however, seem to vary by institutional characteristics even in the same geographic region. Understanding the patterns of prevalence of homelessness is a step toward considering possible interventions to assist this vulnerable population.

## Figures and Tables

**Table 1:** Site characteristics (% payer mix and admission) in study examining prevalence of homelessness in the emergency department setting.

Site	Site A (%)	Site B (%)	Site C (%)
Auto	1.18	2.4	1.43
Blues	8.06	22.08	20.85
Commercial	4.86	11.81	11.50
Medicaid	57.07	14.68	22.61
Medicare	13.42	42.04	36.20
Other	.23	.34	.34
Self-pay	14.41	4.69	5.7
Worker’s comp	.78	1.89	1.33
Admission rates	6.02	32.29	20.60

*Blues,* Blue Cross and Blue Shield insurance; *Auto,* automobile; *Worker’s comp*; worker’s compensation.

**Table 2:** Prevalence of homelessness and “at risk for homelessness” by study site.

Clinical site	At risk n (%)	Homeless n (%)	Total at risk or homeless n (%)	OR (95% CI)	p-value
Site A (n=793)	35 (4.4%)	114 (14.4%)	149 (18.8%)	2.9 (2.2–3.7)	<0.001
Site B (n=1,939)	52 (2.7%)	93 (4.8%)	145 (7.5%)	1.0 (referent)	--
Site C (n=1,663)	49 (2.9%)	102 (6.1%)	151 (9.1%)	1.2 (1.0–1.6)	0.08
Overall	136 (3.1%)	309 (7.0%)	445 (10.1%)		

**Table 3:** Distribution of survey responses by clinical enrollment site.

Survey question	Clinical enrollment site	Survey response		OR (95% CI)*	p-value

		Yes	No		
	
		n(%)	n(%)		
Change in residence	Site A	29 (3.7)	764 (96.3)	2.7 (1.54–4.8)	0.001
	Site B	22 (1.1)	1,917 (98.9)		
	Site C	24 (1.4)	1,639 (98.6)	1.2 (0.7–4.9)	0.48
	Total	75 (1.7)	4,320 (98.3)		
Been concerned about losing house	Site A	81 (10.2)	712 (89.8)	2.3 (1.7–3.2)	<0.001
	Site B	82 (4.2)	1,857 (95.8)	referent	--
	Site C	92 (5.5)	1,571 (94.5)	1.3 (0.9–1.8)	0.09
	Total	255 (5.8)	4,140 (94.2)		
Lived with a family member	Site A	87 (11.0)	706 (89.0)	2.8 (2.0–4.0)	<0.001
	Site B	59 (3.0)	1,880 (97.0)	referent	--
	Site C	75 (4.5)	1,588 (95.5)	1.4 (1.0–2.0)	0.06
	Total	221 (5.0)	4,174 (95.0)		
Been evicted or served eviction	Site A	26 (3.3)	767 (96.7)	3.0 (1.6–5.7)	0.001
	Site B	17 (0.9)	1,922 (99.1)	referent	--
	Site C	23 (1.4)	1,640 (98.6)	1.5 (0.8–2.8)	0.2
	Total	66 (1.5)	4,329 (98.5)		
Slept outside or in abandoned building	Site A	38 (4.8)	755 (95.2)	3.1 (1.8–5.4)	<0.001
	Site B	23 (1.2)	1,916 (98.8)	referent	--
	Site C	20 (1.2)	1,643 (98.8)	1.0 (0.5–1.8)	0.9
	Total	81 (1.8)	4,314 (98.2)		

* All odds ratio estimates adjusted for participant age and gender.

**Table 4:** Distribution of survey questions by time of survey administration (weekday or weekend).

Survey question	Coding	Survey administration timing			OR (95% CI)*	p-value

		Weekday	Weekend	Total		
	No	2,990 (98.4)	1,330 (98.1)	4,320 (98.3)		
	Yes	49 (1.6)	26 (1.9)	75 (1.7)	1.3 (0.8–2.0)	0.37
Been concerned about losing house	No	2,863 (94.2)	1,277 (94.2)	4,140 (94.2)		
	Yes	176 (5.8)	79 (5.8)	255 (5.8)	1.0 (0.8–1.4)	0.76
Lived with a family member	No	2,886 (95.0)	1,288 (95.0)	4,174 (95.0)		
	Yes	153 (5.0)	68 (5.0)	221 (5.0)	1.0 (0.8–1.4)	0.88
Been evicted or served eviction	No	2,993 (98.5)	1,336 (98.5)	4,329 (98.5)		
	Yes	46 (1.5)	20 (1.5)	66 (1.5)	1.0 (0.6–1.7)	0.97
Slept outside or in abandoned building	No	2,980 (98.1)	1,334 (98.4)	4,314 (98.2)		
	Yes	59 (1.9)	22 (1.6)	81 (1.8)	0.9 (0.5–1.4)	0.6

* All odds ratio estimates adjusted for participant age, gender and survey administration location.

**Table 5:** Distribution of survey questions by season.

Survey question	Coding	Season of survey administration			OR (95% CI)*	P-value

		Winter	Summer	Total		
Change in Residence	No	2,288 (98.6)	2,032 (98.0)	4,320 (98.3)		
	Yes	33 (1.4)	26 (2.0)	75 (1.7)	1.2 (0.8–2.0)	0.35
Been concerned about losing house	No	2,201 (94.8)	1,939 (93.5)	4,140 (94.2)		
	Yes	120 (5.2)	135 (6.5)	255 (5.8)	1.2 (0.9–1.5)	0.28
Lived with a family member	No	2,211 (95.3)	1,963 (94.6)	4,174 (95.0)		
	Yes	110 (4.7)	111 (5.4)	221 (5.0)	1.0 (0.7–1.3)	0.84
Been evicted or served eviction	No	2,286 (98.5)	2,043 (98.5)	4,329 (98.5)		
	Yes	35 (1.5)	31 (1.5)	66 (1.5)	0.9 (0.5–1.4)	0.54
Slept outside or in abandoned building	No	2,286 (98.5)	2,028 (97.8)	4,314 (98.2)		
	Yes	35 (1.5)	46 (2.2)	81 (1.8)	1.2 (0.8–1.9)	0.36

* All odds ratio estimates adjusted for participant age, gender and survey administration location.

## References

[1] US Department of Housing and Urban Development (2015). The 2015 Annual Homeless Assessment Report to Congress.

[2] LaCalle E, Rabin E, Genes N (2013). High-frequency users of emergency department care. J Emerg Med.

[3] Hwang SW, Weaver J, Aubry T (2011). Hospital costs and length of stay among homeless patients admitted to medical, surgical, and psychiatric services. Med Care.

[4] Morris DM, Gordon JA (2006). The role of the emergency department in the care of homeless and disadvantage populations. Emerg Med Clin N Am.

[5] Tsai M, Weintraub R, Gee L (2005). Identifying homelessness at an urban public hospital: a moving target?. J Health Care Poor Underserved.

[6] Ku B, Scott K, Kertesz S (2010). Factors associated with use of urban emergency departments by the U.S. homeless population. Public Health Rep.

[7] (2011). Current Statistics on the Prevalence and Characteristics of People Experiencing Homelessness in the United States.

[8] Chambers C, Chiu S, Hwang S (2013). High utilizers of emergency health services in a population-based cohort of homeless adults. Am J Public Health.

[9] Oates G, Tadros A, Davis SM (2009). A comparison of national emergency department use by homeless versus non-homeless people in the United States. J Health Care Poor Underserved.

[10] Doran KM, Vashi AA, Platis S (2013). Navigating the boundaries of emergency department care: addressing the medical and social needs of patients who are homeless. Am J Public Health.

[11] Bon SK, Fields JM, Santana A (2014). The urban homeless: Super utilizers of the Emergency Department. Pop Health Management.

[12] (2015). Homeless Emergency Assistance and Rapid Transition to Housing (HEARTH): Defining “Chronically Homeless” Final Rule. U.S. Department of Housing and Urban Development Exchange website.

[13] (2011). Homeless Emergency Assistance and Rapid Transition to Housing: Defining “Homeless”. Department of Housing and Urban Development.

[14] Department of Veterans Affairs Office of Inspector General (2012). Homeless Incidence and risk factors for becoming homeless in veterans.

[15] The State of Homelessness in America.

[16] Brown AJ, Goodacre SW, Cross S (2010). Do emergency department attendances by homeless people increase in cold weather?. Emerg Med J.

[17] Williams M, Cheal B (2002). Can we measure homelessness? A critical evaluation of ‘Capture-Recapture’. Int J Soc Res Meth.

